# Structural and Functional Analysis of the Pyridoxal Phosphate Homeostasis Protein YggS from *Fusobacterium nucleatum*

**DOI:** 10.3390/molecules27154781

**Published:** 2022-07-26

**Authors:** Shanru He, Yuanyuan Chen, Lulu Wang, Xue Bai, Tingting Bu, Jie Zhang, Ming Lu, Nam-Chul Ha, Chunshan Quan, Ki Hyun Nam, Yongbin Xu

**Affiliations:** 1 Department of Bioengineering, College of Life Science, Dalian Minzu University, Dalian 116600, China; heshanru315@gmail.com (S.H.); chenyuanyuan@meilunbio.com (Y.C.); wanglulu0813@126.com (L.W.); baixue201609@126.com (X.B.); butingting2020@126.com (T.B.); j_zhang1128@163.com (J.Z.); 2 Key Laboratory of Biotechnology and Bioresources Utilization of Ministry of Education, College of Life Science, Dalian Minzu University, Dalian 116600, China; 3 School of Life Science and Biotechnology, Dalian University of Technology, No. 2 Linggong Road, Dalian 116024, China; 4 Shandong Provincial Key Laboratory of Energy Genetics, Key Laboratory of Biofuel, Qingdao Institute of Bioenergy and Bioprocess Technology, Chinese Academy of Sciences, Qingdao 266101, China; lvming@qibebt.ac.cn; 5 Department of Agricultural Biotechnology, College of Agriculture and Life Sciences, Seoul National University, Gwanak-gu, Seoul 00826, Korea; hanc210@snu.ac.kr; 6 Department of Life Science, Pohang University of Science and Technology, Pohang 37673, Korea; 7 POSTECH Biotech Center, Pohang University of Science and Technology, Pohang 37673, Korea

**Keywords:** pyridoxal 5′-phosphate, PLP, YggS, *Fusobacterium nucleatum*, crystal structure

## Abstract

Pyridoxal 5′-phosphate (PLP) is the active form of vitamin B6, but it is highly reactive and poisonous in its free form. YggS is a PLP-binding protein found in bacteria and humans that mediates PLP homeostasis by delivering PLP to target enzymes or by performing a protective function. Several biochemical and structural studies of YggS have been reported, but the mechanism by which YggS recognizes PLP has not been fully elucidated. Here, we report a functional and structural analysis of YggS from *Fusobacterium nucleatum* (FnYggS). The PLP molecule could bind to native FnYggS, but no PLP binding was observed for selenomethionine (SeMet)-derivatized FnYggS. The crystal structure of FnYggS showed a type III TIM barrel fold, exhibiting structural homology with several other PLP-dependent enzymes. Although FnYggS exhibited low (<35%) amino acid sequence similarity with previously studied YggS proteins, its overall structure and PLP-binding site were highly conserved. In the PLP-binding site of FnYggS, the sulfate ion was coordinated by the conserved residues Ser201, Gly218, and Thr219, which were positioned to provide the binding moiety for the phosphate group of PLP. The mutagenesis study showed that the conserved Ser201 residue in FnYggS was the key residue for PLP binding. These results will expand the knowledge of the molecular properties and function of the YggS family.

## 1. Introduction

*Fusobacterium nucleatum* is an anaerobic gram-negative bacterium that plays a key role in oral pathological conditions [1,2,3]. It has frequently been associated with a wide spectrum of human diseases [4,5]. Recent studies have shown that *F. nucleatum* is a causative agent of appendicitis, gingivitis, osteomyelitis, and pregnancy complications, leading to increasing attention in this species [5,6,7,8,9,10,11,12,13]. Interestingly, it is associated with colorectal cancer (CRC) progression via its ability to activate the autophagy pathway in CRC, and it is linked to the progression and severity of CRC and chronic inflammatory periodontitis [14,15]. *F. nucleatum* has been identified as an urgent threat to human health [16,17,18]. Considering the increasing antibiotic resistance of *F. nucleatum*, the development of new drugs is urgently needed [19,20].

The physiologically active form of vitamin B6, pyridoxal 5′-phosphate (PLP), is an essential cofactor for dozens of bacterial and hundreds of human enzymes that are involved in diverse cellular processes [21,22,23,24]. YggS, a PLP-dependent enzyme that is involved in cell wall metabolism and information processing, belongs to the COG0325 gene family in bacteria [25]. The pyridoxine toxicity phenotype and amino acid-related metabolic disorders are caused by the inactivity of YggS [26]. The PLP-binding proteins are cotranscribed with proline biosynthesis genes; hence, this group of proteins is also named the proline synthetase cotranscribed homolog (PROSC) family [27]. Several crystal structures of YggS proteins from *Synechococcus elongatus* (Protein Data Bank (PDB) ID: 5NM8, named SePipY) [28], *Saccharomyces cerevisiae* (1B54, ScP007) [29], *Escherichia coli* (1W8G, EcYggS; unpublished), and *Bifidobacterium adolescentis* (3CPG, BaPipY; unpublished) are currently deposited in the PDB. The above proteins contain the typical type III fold of PLP-dependent enzymes [30,31], and these structural studies have provided fundamental information on how YggS recognizes PLP molecules.

Aiming to define the role of YggS, we identified a protein annotated as YggS in the *F. nucleatum* genome (UniProt accession no. Q8RFW9); this protein exhibited less than 35% sequence similarity with previously reported YggS proteins. Therefore, we considered *F. nucleatum* YggS (abbreviated FnYggS) to be a potential target. Understanding the potential molecular mechanisms of the FnYggS protein is thus important for the development of anti-*F. nucleatum* drugs. Although considerable effort is being devoted to understanding the action mechanism of YggS proteins, research on the regulation of PLP levels and the means by which PLP is transferred from the product to the catalytic site is challenging [32].

To improve the understanding of the molecular function of YggS, we report a structural and functional analysis of FnYggS. Moreover, to understand the molecular interaction between FnYggS and the YggS-interacting protein SepF, we performed a pull-down assay. Our results can provide valuable information for improving the understanding of the molecular functions of PLP-binding proteins and elucidating the binding properties.

## 2. Results and Discussion

### 2.1. Characterization of FnYggS

**Figure 1:**
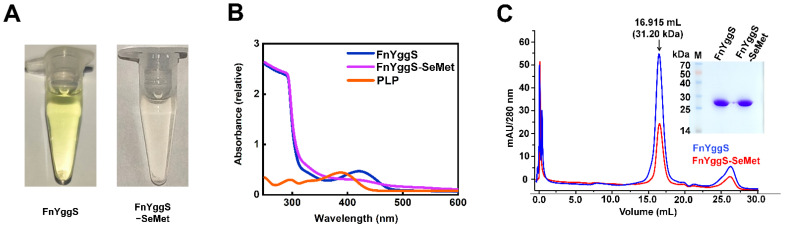
Characterization of PLP binding to FnYggS. (**A**) The FnYggS and FnYggS-SeMet solutions were yellow and colorless, respectively. (**B**) Native FnYggS exhibited an absorption peak at 425 nm due to PLP binding, whereas FnYggS-SeMet did not exhibit an absorption peak, indicating the absence of PLP binding. The absorption peak of PLP was seen at 380 nm. (**C**) Purified native and SeMet-derivatized FnYggS solutions and size exclusion chromatography results. Both proteins existed as monomers in solution.

After the purification of native FnYggS, the concentrated FnYggS solution exhibited a yellow color. Since free PLP is yellowish, with an absorbance peak at 380 nm [27], we concluded that the PLP produced in *E. coli* may bind to FnYggS (Figure 1A). To verify the binding of PLP to FnYggS, the purified native FnYggS solution was subjected to spectroscopic analysis. The native FnYggS showed an absorption peak at 425 nm, indicating that PLP was bound to FnYggS (Figure 1B). Free PLP showed an absorption peak at 380 nm, as previously reported [27]. On the other hand, the absorbance peak of fresh PLP-bound *S. elongatus* PipY (SePipY) was observed at 425 nm [27]. Taken together, these findings indicate that PLP-bound YggS proteins exhibit absorption peaks at approximately 420–425 nm, but the maximum absorption peak of each YggS occurs at a distinct wavelength.

Meanwhile, in the crystallographic study, we substituted the methionine residues of FnYggS with SeMet (abbreviated FnYggS-SeMet) to address the phasing problem by using the Se-single-wavelength anomalous diffraction (Se-SAD) method. Interestingly, the purified FnYggS-SeMet solution was not yellow (Figure 1A), indicating that FnYggS-SeMet most likely did not bind PLP. To verify the absence of PLP binding, the absorbance of purified FnYggS-SeMet was measured. A very low absorption peak corresponding to the PLP molecule was observed at 425 nm, indicating a very weak interaction of PLP with FnYggS-SeMet (Figure 1B). We concluded that the presence of SeMet around the PLP binding site interferes with PLP binding (see below).

Next, to determine whether the oligomerization state of FnYggS is influenced by PLP, purified FnYggS (PLP-bound state) and FnYggS-SeMet (PLP-unbound state) were analyzed by analytical gel filtration chromatography (Figure 1C). Both native FnYggS and FnYggS-SeMet existed as monomers in solution (Figure 1C). These results demonstrate that PLP does not impact the oligomeric state of FnYggS.

### 2.2. Overall Structure of FnYggS

To better understand the properties underlying PLP recognition, we determined the crystal structure of FnYggS at 2.08 Å resolution by Se-SAD. The crystal structure belonged to the monoclinic space group P2_1_ with a = 37.929 Å, b = 146.375 Å, c = 74.128 Å, α = γ = 90° and β = 93.355°. The *R*_factor_ and *R*_free_ of the final FnYggS model were 18.39% and 21.77%, respectively (Table 1).

**Figure 2:**
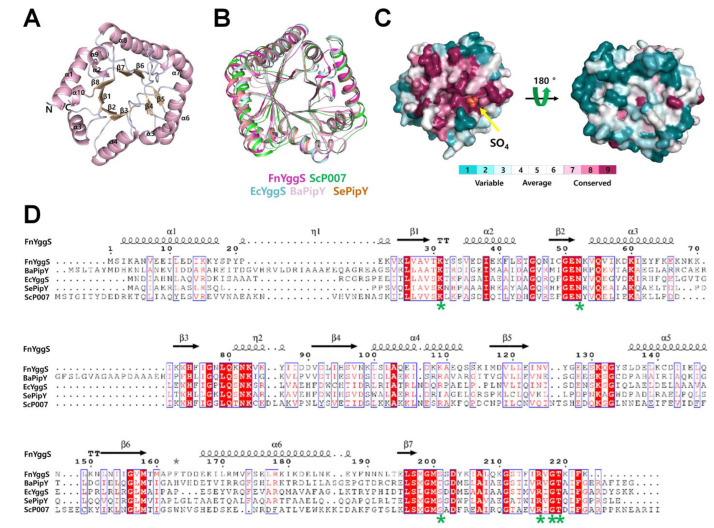
Crystal structure of FnYggS. (**A**) Cartoon representation of the FnYggS monomer consisting of a typical TIM barrel fold. (**B**) Superimposition of FnYggS (purple, PDB ID: 7YGF) onto ScP007 (green, PDB ID: 1B54), EcYggS (blue, 1W8G), BaPipY (pink, 3CPG), and SePipY (brown, 5NM8). (**C**) Surface conservation of PipY proteins. The PLP-binding site showed high conservation, whereas other regions were not conserved. The PLP-binding site is indicated by the yellow arrow. (**D**) Sequence alignment of FnYggS (UniProt accession no. Q8RFW9) with BaPipY (A1A3G9), EcYggS (P67080), SePipY (Q31LH9), and ScP007 (P38197). The residues forming the PLP-binding site are indicated by the green asterisks.

The electron density map showed a clear electron density for all of the amino acid residues from Met1 to Lys223 in the A chain and C chain, while in the B chain of FnYggS, most amino acid residues from Met1 to Lys223 showed a clear electron density, except for Glu128-Gln132. FnYggS consists of 8 β-strands and 10 α-helices and forms a TIM barrel fold typical of the type III fold of PLP-dependent enzymes (Figure 2A).

The DaliLite server was used to search for structural homologs of the FnYggS protein [31]. FnYggS exhibited structural similarity with *S. elongatus* PipY (Z score = 32.3), EcYggS (31.0), *B. adolescentis* PipY (29.0), and *S. cerevisiae* PipY (27.7). Although FnYggS shared low sequence identities with SePipY (sequence identity: 33.18%), EcYggS (31.05%), AfPipY (30.19%), BaPipY (34.86%), and ScP007 (31.96%), the superimposition of FnYggS with SePipY, EcYggS, AfPipY, BaPipY, and ScP007 revealed high structural similarity, with an r.m.s. deviation of 1.3–2.0 Å (Figure 2B). Conserved surface and sequence alignment of YggS proteins showed that the PLP-binding site was highly conserved, whereas other regions had no amino acid conservation (Figure 2C,D).

### 2.3. PLP-Binding Site of FnYggS

**Figure 3:**
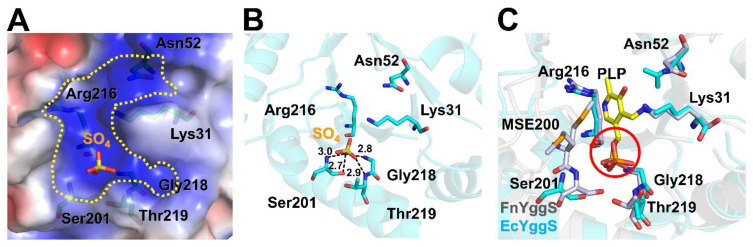
PLP-binding site and mutagenesis of FnYggS. (**A**) Electrostatic surface of the PLP-binding site of FnYggS. (**B**) The sulfate ion in the PLP-binding pocket of FnYggS is coordinated by Ser201, Gly218, and Thr219. (**C**) Superimposition of the PLP-binding site of FnYggS (purple) onto PLP-bound EcYggS (cyan, PDB ID: 1W8G).

The PLP-binding pocket is formed by six amino acids (Lys31, Asn52, Ser201, Gly218, Arg216, and Thr219), which are highly conserved in YggS family members (Figure 2D) and form a positively charged surface (Figure 3A). The crystal structure of FnYggS-SeMet did not exhibit the electron density considered to represent the PLP molecule, consistent with our spectroscopy experiments showing the absence of PLP binding in FnYggS-SeMet. Instead, a sulfate ion was observed in the PLP-binding site of FnYggS. This sulfate ion was coordinated by the hydroxyl oxygen atom of Ser201 (2.7 Å), the nitrogen atom of Ser201 (3.0 Å), the nitrogen atom of Gly218 (2.8 Å), and the nitrogen atom of Thr219 (2.9 Å) (Figure 3B). Interestingly, the position of the sulfate ion in FnYggS-SeMet was almost the same as that of the phosphate group of PLP in the EcYggS structure (Figure 3C). This result indicates that the sulfate ion in FnYggS-SeMet is positioned to provide the binding moiety, as is the phosphate group in PLP. On the other hand, PLP was not bound to FnYggS-SeMet (Figure 1). These results indicate that the substitution of the SeMet residue interferes with the binding of PLP. FnYggS contains seven methionine residues, among which Met200 is located at the PLP-binding site and is downstream of Ser201, which is involved in PLP binding. During structural refinement, the side chain of SeMet200 showed two distinct conformations (Figure 3C). In particular, one methionine residue was oriented toward the PLP-binding pocket (Figure 3C).

**Figure 4:**
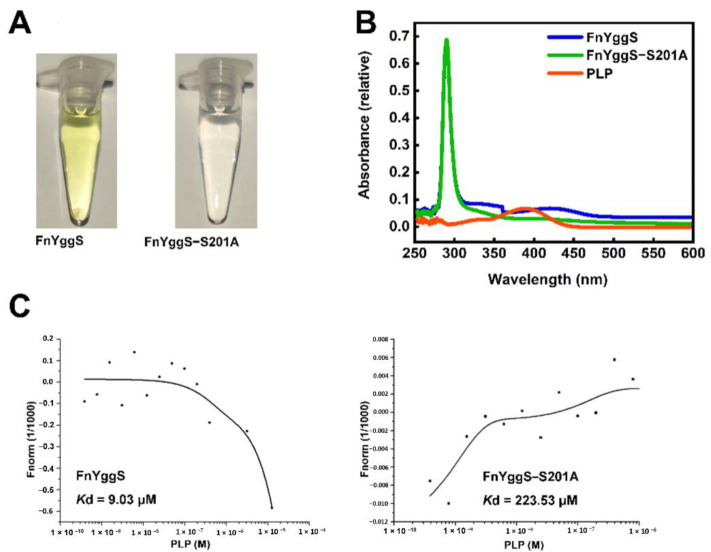
(**A**) Purification and (**B**) spectroscopic analysis of FnYggS (blue) and FnYggS-S201A (green), indicating that Ser201 is critical for PLP binding. (**C**) The binding affinity of FnYggS or FnYggS-S201A and PLP was measured by MST.

Among the phosphate-binding residues, Ser201 is a conserved amino acid in YggS proteins (Figure 2D), and compared to other residues, it is tightly bound to the sulfate group (Figure 3B). Ser201 of FnYggS was substituted with an alanine residue (abbreviated FnYggS-S201A) through mutagenesis to determine whether Ser201 of FnYggS is responsible for PLP binding. The purified FnYggS-S201A solution was not yellow, indicating that PLP was not bound (Figure 4A). To verify the absence of PLP binding, spectroscopic analysis was performed. The absorbance peak at approximately 420 nm indicated that bound PLP was not observed for FnYggS-S201A (Figure 4B), indicating that the conserved Ser201 residue of FnYggS is a key residue for PLP binding.

To confirm the binding affinity of PLP with FnYggS, FnYggS-S201A, and FnYggS-SeMet, microscale thermophoresis (MST) assays were used for quantitative analysis. The MST results revealed that wild-of FnYggS bound to PLP with a *Kd* of 9.03 μM; however, the binding affinity values of PLP with FnYggS-S201A were significantly decreased, with a *Kd* value of 223 μM (Figure 4C). The results indicated that FnYggS could bind to the PLP ligand and that Ser201 played crucial roles in the binding of PLP and FnYggS. These results were consistent with the spectroscopic analysis results mentioned above.

**Figure 5:**
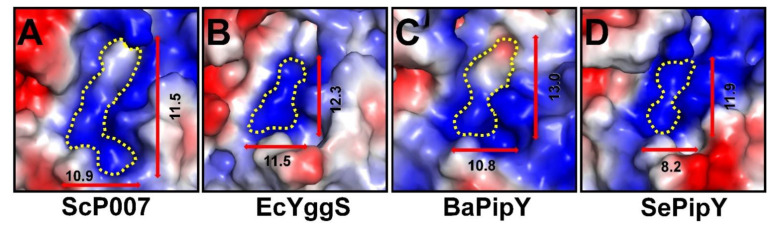
Electrostatic surface of the PLP-binding pocket of (**A**) ScP007 (PDB ID: 1B54), (**B**) EcYggS (1W8G), (**C**) BaPipY (3CPG), and (**D**) SePipY (5NM8).

Next, the PLP-binding site of FnYggS was compared with those in ScP007 (PLP-bound state), EcYggS (PLP-bound state), SePipY (PLP-bound state), and BaPipY (PLP-unbound state). The binding pockets of YggS proteins are commonly positively charged, but the PLP-binding pocket of each YggS protein has not only a unique shape but also a distinct size (Figure 5). In the PLP-unbound state, FnYggS contains a wider PLP-binding pocket than other YggS proteins, but we concluded that the size of the PLP-binding pocket in FnYggS is changed upon binding of the PLP molecule.

## 3. Materials and Methods

### 3.1. Protein Preparation

The genomic DNA of *F. nucleatum* was used as a template. The gene encoding FnYggS (UniProt: A0A117MW82, residues 1–223) was cloned into the expression vector pET-28a(+) (Invitrogen, Waltham, MA, USA) at the *Nco*I and *Xho*I restriction sites. The expression and purification of FnYggS have been previously described [32]. Site-directed mutagenesis for FnYggS-S201A was performed using two subsequent PCRs. The mutant sequence was confirmed through DNA sequencing, and the expression and purification of the recombinant protein were found to be the same as those of native FnYggS.

### 3.2. Analysis of PLP Binding

PLP binding to FnYggS was verified by absorbance measurements using ultraviolet–visible (UV-Vis-2450) spectroscopy (Shimadzu Corporation). Native FnYggS (0.15 mM), SeMet-derivatized FnYggS (0.15 mM), or FnYggS-S201A (0.15 mM) (200 μL each) were added to cuvettes, and the absorbance was measured at room temperature across the wavelength range of 250–600 nm.

### 3.3. Crystallization, Data Collection, and Structural Determination

Crystallization and preliminary X-ray analysis of FnYggS have been published previously [32]. The structure of FnYggS was determined by single-wavelength anomalous diffraction (SAD). X-ray diffraction datasets were collected on beamline 5C at Pohang Light Source II (PLS-II, Pohang, Republic of Korea) at −173.15 °C. The data were then indexed, integrated, and scaled using the HKL2000 software suite [33]. The visualization of the electron density maps and the manual rebuilding of the atomic model were performed using the program COOT [34]. Refinement was performed using the PHENIX package [35]. Then, cycles of structural refinement were carried out using PHENIX refinement in the PHENIX package [35]. The geometry of the final model was validated with MolProbity [36]. The refinement statistics are provided in Table 1. Surface conservation within the YggS family of proteins was calculated using the Consurf server [37]. All structural figures were generated with PyMOL [38]. Multiple sequence alignment was performed using the Clustal Omega server [39].

### 3.4. Size Exclusion Chromatography

The oligomeric state of FnYggS was assessed by performing size exclusion chromatography with a Superdex 200 10/600 GL column (GE Healthcare, Chicago, IL, USA). A microsyringe was used to inject 500 μL of purified FnYggS (0.5 mg mL^−1^) into the column. The column was pre-equilibrated with a column buffer consisting of 20 mM HEPES (pH 8.0), 150 mM NaCl, and 2 mM BME, and the absorbance of the protein was monitored at 280 nm. The molecular weights of the eluted samples were calculated based on the calibration curves by several standard samples (Gel Filtration Markers Kit for Protein Molecular Weights 12,000–200,000 Da, Sigma–Aldrich, St. Louis, MO, USA), such as cytochrome c, carbonic anhydrase, albumin, alcohol dehydrogenase, β-amylase, and blue dextran.

### 3.5. Mutagenesis

The plasmids used for the expression of the FnYggS mutants, which were used in the analysis of PLP binding, were constructed by two subsequent PCRs. The first round of PCR was used to amplify the upstream mutated segment using the forward primer F 5′-GGG CCA TGG GCC ACC ATC ACC ATC ACC ATA TGA GTA TAA AAG CAA ATG TTG AAG-3′ and the reverse primer 5′-TAT CTT ATA ATC ACT AGC CAT TCC CAT TGA AAG-3′. The first round of PCR was used to amplify the downstream mutated segment using the forward primer 5′-CTT TCA ATG GGA ATG GCT AGT GAT TAT AAG ATA-3′ and the reverse primer R 5′-GGG CTC GAG TTA TTT AAA AAT TTT TGT TCC AAC-3′. The second round of PCR introduced an overhang using DNA fragments generated in the first round of PCR as templates and the primers F and R, and they were cloned into the expression vector pET-28a(+) (Invitrogen, USA) at the *Nco*I and *Xho*I restriction sites. Furthermore, the plasmids were transformed into *E. coli* BL21 (DE3) cells that were cultured in Luria-Bertani (LB) medium. The FnYggS mutant was expressed and purified by a method similar to that used for native FnYggS.

### 3.6. Microscale Thermophoresis

The affinities of the YggS and YggS variants for the ligand PLP were measured by MST. Briefly, the binding reactions contained 20 mM Tris-HCl (pH 7.5), 150 mM NaCl, 10 mM MgCl_2_, and 0.05% (*v*/*v*) Tween-20 in a total volume of 20 μL. The FnYggS and FnYggS-S201A proteins without PLP were cultured in M9 medium, using methionine instead of L-(+)-selenomethionine (SeMet). For the binding reaction, different concentrations of ligand (PLP) and a constant concentration of YggS labeled with RED-NHS (Monolith^TM^ Series Protein Labeling Kit RED-NHS 2nd Generation, Nanotemper) were used. MST analysis was performed using standard capillaries from Nanotemper. MST analysis was performed on a Monolith NT.115 instrument (Nanotemper Technologies) using 20% LED and 40% MST power at room temperature. The *Kd* was calculated by taking the average of triplicate F norm measurements at each concentration from three independent MST measurements. Data analyses were performed using Nanotemper Analysis software, v 2.3.

## Figures and Tables

**Table 1:** Data collection and refinement statistics for DendFP.

Data Collection	FnYggS
Diffraction source	Beamline 5A, PLS-II
Wavelength	0.9793
Detector	ADSC Q315r CCD
Rotation range per image (°)	1
Total rotation range (°)	360
Exposure time per image (s)	0.5
Space group	P2_1_
Cell dimensions	
a, b, c (Å)	37.929, 146.375, 74.128
Resolution (Å)	36.59–2.08
Completeness	96.03 (86.73)
Redundancy	4.2 (2.7)
I/σ(I)	27.1304 (4.33)
Rsym (%)	27.1 (60.7)
Refinement statistics	
R_work_ (%)	18.39 (19.59)
R_free_ (%)	21.77 (22.86)
B-factor (Averaged)	
Protein	28.83
R.m.s. deviations	
Bond lengths (Å)	0.009
Bond angles (°)	1.23
Ramachandran plot (%)	
favored	97.59
allowed	2.26
disallowed regions	0.15
PDB code	7YGF

Values in parentheses are for the outermost shell.

## Data Availability

The atomic coordinates and structural factors for FnYggS (PDB ID: 7YGF) have been deposited in the Protein Data Bank.

## Sample Availability

Samples of the compounds are not available from the authors.
